# Local Integral Estimates for Quasilinear Equations with Measure Data

**DOI:** 10.1155/2016/5742063

**Published:** 2016-05-17

**Authors:** Qiaoyu Tian, Shengzhi Zhang, Yonglin Xu, Jia Mu

**Affiliations:** ^1^School of Mathematics and Computer, Northwest University for Nationalities, Lanzhou, Gansu 730030, China; ^2^Department of Mathematics, Gansu Normal University for Nationalities, Hezuo, Gansu 747000, China

## Abstract

Local integral estimates as well as local nonexistence results for a class of quasilinear equations −Δ_*p*_ *u* = *σP*(*u*) + *ω* for *p* > 1 and Hessian equations *F*_*k*_[−*u*] = *σP*(*u*) + *ω* were established, where *σ* is a nonnegative locally integrable function or, more generally, a locally finite measure, *ω* is a positive Radon measure, and *P*(*u*) ~ exp⁡*αu* ^*β*^ with *α* > 0 and *β* ≥ 1 or *P*(*u*) = *u* ^*p*−1^.

## 1. Introduction and Main Results

Let *Ω* ⊂ *ℝ* ^*N*^ be a bounded domain, let *σ* be a nonnegative locally integrable function or, more generally, a locally finite measure on *Ω*, and let *ω* be a nonnegative Borel measure. In this paper, we consider the following nonlinear partial differential equations with measure data:

(1) −Δpu=σPu+ω,u≥0,  u  is  p-superharmonic  in  Ω,

where *P*(*u*) = *P*_*ℓ*,*α*,*β*_(*u*) ∈ *L*_*σ*,loc_^1^(*Ω*) is defined, following [1, 2], as

(2) $$\begin{array}{c} P_{l,\alpha ,\beta }\left(\;u\;\right)=H_{l}\left(\;\alpha u^{\beta }\;\right) \end{array}$$

and *ℓ*-truncated exponential function *H*_*ℓ*_(*r*) is given by

(3) $$\begin{array}{c} H_{l}\left(\;r\;\right)=\overset{\infty }{\underset{j=l}{\sum }}\frac{r^{j}}{j!}. \end{array}$$

Here, Δ_*p*_ *u* is the *p*-Laplacian of *u* defined by Δ_*p*_ *u*≔div⁡(|*Du*|^*p*−2^ *Du*)  (*p* > 1). For convenience, here and elsewhere in the paper, we assume that *ℓβ* > *p* − 1. We will understand (1) in the following potential-theoretic sense using *p*-superharmonic functions (see Section 2).

Quasilinear equations (1) where *σP*(*u*) term is replaced by *σu* ^*q*^ for superlinear case *q* > *p* − 1 are well studied (see [3–5] and references therein, see [6, 7] for natural growth terms *q* = *p* − 1, and see [8, 9] for sublinear problems *q* < *p* − 1). In particular, it was shown in [3] that if *u* ∈ *L*_*σ*,loc_^*q*^(*Ω*) is a solution of (1) with nonlinear source terms *σu* ^*q*^, *q* > *p* − 1 in *Ω* and *B*_4*R*_ ⊂ *Ω*, then there is a constant *M* = *M*(*N*, *p*, *q*) such that

(4) ∫0rσBttN−p1/p−1dttp−1/q−p+1·∫rR/2∫BtuqdσtN−p1/p−1dtt≤M,

for all 0 < *r* ≤ *R*/4; (4) improves the related results of [10, 11].

Recently, Quoc-Hung and Véron [2] obtained two-sided estimates on the solutions in terms of *T*-truncated *η*-fractional maximal potential of *ω*, which is suitable for dealing with exponential nonlinearities:

(5) $$\begin{array}{c} M_{\alpha ,T}^{\eta }=\sup\left\{\;\frac{\omega \left(B_{t}\right)}{t^{N-\alpha }h_{\eta }\left(t\right)}:0<t\leq T\;\right\}, \end{array}$$

where

(6) hηt=−ln⁡t−ηχ0,1/2t+ln⁡2−ηχ1/2,+∞t,η>0,1,η=0.

Some analogous estimates for Hessian equations also are given in this paper.

In this paper, firstly, we will establish a priori estimates of (1) with exponential reaction *P*_*ℓ*,*α*,*β*_(*u*), defined by (2) and (3). One of our main results are the following theorems.

**Theorem 1 .**

Let *u*(*x*) be a solution of (1) in *Ω* with *p* > 1 and *ℓβ* > *p* − 1. Suppose that *B*_4*R*_(*x*_0_) ⊂ *Ω*. Then, there exists a constant *M* = *M*(*N*, *p*, *α*, *β*, *ℓ*) such that

(7) ∫0rσBttN−p1/p−1dttp−1/lβ−p+1·∫rR/2ωBtx0tN−p1/p−1dtt≤M,

(8) ∫0rσBttN−p1/p−1dttp−1/lβ−p+1·∫rR/2∫BtPl,α,βudσtN−p1/p−1dtt≤M,

for all 0 < *r* ≤ *R*/4.

As a consequence of Theorem 1, we have the following nonexistence results of local solutions to quasilinear equations.

**Theorem 2 .**

Let *u* be a solution of (1) in an open connected set *Ω* ⊂ *ℝ* ^*N*^. Suppose that 1 < *p* < *n*, *dσ* = |*x*|^−*r*^ *dx* with *r* > *p*, and 0 ∈ *Ω*. Then, *u* ≡ 0.

Now, we consider (1) with natural growth terms; that is, the *σP*(*u*) term in (1) is replaced by *σu* ^*p*−1^. It is worthwhile to point out that this problem turns out to be more complex than the supercritical case. The interaction between the differential operator −Δ_*p*_ *u* and the lower order term *σu* ^*p*−1^ was investigated by Jaye and Verbitsky [6, 7].

**Theorem 3 .**

Let *u*(*x*) be a solution of (1) in *Ω* with *p* > 1 and *P*(*u*) = *σu* ^*p*−1^. Suppose that *B*_4*R*_(*x*_0_) ⊂ *Ω*. Then, there exists a constant *M* = *M*(*N*, *p*, *α*, *β*, *ℓ*) such that

(9) ∫0rσBttN−p1/p−1dtt∫rR/2ωBtx0tN−p1/p−1dtt≤M,

(10) ∫0rσBttN−p1/p−1dtt∫rR/2∫Btup−1dσtN−p1/p−1dtt≤M,

for all 0 < *r* ≤ *R*/4.

Similarly, we have the following.

**Theorem 4 .**

Let *u* ∈ *L*_*loc*_^*p*−1^(*dσ*) be a solution of (1) with *P*(*u*) = *σu* ^*p*−1^ in an open connected set *Ω* ⊂ *ℝ* ^*N*^. Suppose that 1 < *p* < *n*, *dσ* = |*x*|^−*p*^ *dx*, and 0 ∈ *Ω*. Then, *u* ≡ 0.

**Remark 5 .**

The four previous theorems are particular case of the more general class of nonlinear Wolff integral equations:

(11) $$\begin{array}{c} u\left(\;x\;\right)=W_{\alpha ,p}^{R}\left[\;P\left(\;u\;\right)d\sigma \;\right]\left(\;x\;\right)+f\left(\;x\;\right), \end{array}$$

where *f*(*x*) = **W**_*α*,*p*_^*R*^[*ω*](*x*), which includes fractional Laplacian (−Δ)^*γ*^. Therefore, we also can obtain similar results of these integral equations.

The plan of the paper is as follows. In Section 2, we collect some elements notions and potential estimates for *p*-superharmonic. Theorems 1 and 2 will be proved in Section 3. In this section, we also discuss the relations of *σ* and *ω* provided that there exist solutions of (1). After this, Section 4 presents the proof of Theorems 3 and 4 by a new iteration scheme.  Section 5 is devoted to considering fully nonlinear analogues of the Dirichlet problem (1) for Hessian equations without proof.

## 2. Preliminaries

In this section, we first recall some notations and definitions. In the following, we denote by *C* a general constant, possibly varying from line to line, to indicate a dependence of *C* on the real parameters *N*, *p*, *α*, *β*, *ℓ*; we will write *C* = *C*(*N*, *p*, *α*, *β*, *ℓ*). We also denote by *B*(*x*_0_, *r*) = {*x* ∈ *ℝ* ^*n*^ : |*x* − *x*_0_| < *r*} the open ball with center *x*_0_ and radius *r* > 0; when it is not important or clear from the context, we shall omit denoting the center as *B*_*r*_ = *B*(*x*_0_, *r*).

Let *p* > 0 and *σ* be a nonnegative Borel measures in *Ω* which are finite on compact subsets of *Ω*. The *σ*-measure of a measurable set *E* ⊂ *Ω* is denoted by *σ*(*E*)≔∫_*E*_ *dσ*. We denote by *L* ^*p*^(*Ω*, *dσ*)  (*L*_loc_^*p*^(*Ω*, *dσ*), resp.) the space of measurable functions *f* such that |*f*|^*p*^ is integrable (locally integrable) with respect to *σ*. When *dσ* = *dx*, we write *L* ^*p*^(*Ω*) (*L*_loc_^*p*^(*Ω*), resp.).

For *α* > 0,  *p* > 1, such that *αp* < *n*, the *R*-truncated Wolff's potential **W**_1,*p*_^*R*^ *μ*(*x*) of a nonnegative Borel measure *μ* on *ℝ* ^*N*^ is defined by

(12) $$\begin{array}{c} W_{1,p}^{R}\left[\;\mu \;\right]\left(\;x\;\right)=\overset{R}{\underset{0}{\int }}{\left(\;\frac{\mu \left(B_{t}\right)}{t^{N-p}}\;\right)}^{1/\left(p-1\right)}\frac{dt}{t}. \end{array}$$

We also denote by **W**_1,*p*_[*μ*](*x*) the *∞*-truncated Wolff's potential.

In this paper, all solutions are understood in the potential-theoretic sense. A lower semicontinuous function *u* : *Ω* → (−*∞*, +*∞*] is called *p*-superharmonic if *u* is not identically infinite in each component of *Ω*, and if for all open sets *D* such that $\bar{D}\subset \Omega$, and all functions *h* ∈ *C*(*D*), *p*-harmonic in *D*, the implication holds: *h* ≤ *u* on ∂*D* implies *h* ≤ *u* in *D*. Note that *p*-superharmonic function *u* does not necessarily belong to *W*_loc_^1,*p*^(*Ω*), but its truncation *T*_*k*_(*u*) = min⁡{*u*, *k*} does for every integer *k*; therefore, we will need the generalized gradient of a *p*-superharmonic function *u* defined by *Du* = lim_*k*→*∞*_∇(*T*_*k*_(*u*)). For more properties of *p*-superharmonic, see [12].

The following lower pointwise estimates for *p*-superharmonic functions play an important role in our estimate.

**Proposition 6 (see [13]).**

There exists a positive constant *C* = *C*(*N*, *p*) such that if *u* ≥ 0 is *p*-superharmonic on *Ω* and *μ* = −Δ_*p*_ *u*, then

(13) $$\begin{array}{c} {\left[\;\frac{\mu \left(B_{R}\right)}{R^{N-p}}\;\right]}^{1/\left(p-1\right)}\leq C\underset{B_{R}}{\;\inf}u, \end{array}$$

for all balls *B*_*R*_ such that *B*_2*R*_ ⊂ *Ω*.

The following lemma was also proved in [13].

**Proposition 7 .**

Let *B*_*R*_(*x*) be a ball such that *B*_2*R*_(*x*) ⊂ *Ω*. Then, there exists a positive constant *C* = *C*(*N*, *p*) such that if *u* ≥ 0 is *p*-superharmonic on *Ω* and *μ* = −Δ_*p*_ *u*, then

(14) $$\begin{array}{c} CW_{1,p}^{R}\left[\;\mu \;\right]\left(\;x\;\right)\leq u\left(\;x\;\right), \end{array}$$

where **W**_1,*p*_^*R*^[*μ*](*x*) is the Wolff potential of *μ*.

Given *r* > 0, we consider a ball *B*(*x*, 2*r*) ⊂ *Ω* and shrinking balls *B*_*j*_≔*B*_*r*_*j*__(*x*_0_), where *r*_*j*_ = *r*2^−*j*^ with *j* ≥ 0 is an integer.

**Proposition 8 (see Lemma 2.5 in [3]).**

Let *μ* be locally finite nonnegative measures on *Ω*. Then, there exists a constant *C* = *C*(*N*, *p*) > 0 such that for any *s* > 0 we have

(15) $$\begin{array}{c} \phi^{s+1}\leq \left(\;s+1\;\right)W_{1,p}^{R}\left(\;\phi^{s\left(p-1\right)}d\sigma \;\right), \end{array}$$

where *ϕ* = ∑_*j*=0_^*∞*^ *c*_*j*_ *χ*_*B*_*j*__ with *c*_*j*_ = *C*[*σ*(*B*_*j*_)/(*r*2^−*j*^)^*N*−*p*^]^1/(*p*−1)^.

The following theorem is an analogue of the above theorems for *k*-Hessian equations. For more details, see [14].

**Proposition 9 .**

If *u* ≥ 0 is such that −*u* is *k*-convex in *Ω*, then

(16) $$\begin{array}{c} {\left[\;\frac{\mu \left(B_{R/8}\right)}{R^{N-2k}}\;\right]}^{1/k}\leq C\left(\;n,k\;\right)\underset{B_{R/8}}{\inf}u, \end{array}$$

for all balls *B*_*R*_, provided that *B*_3*R*_ ⊂ *Ω*; here, *μ* = *F*_*k*_[−*u*] is the corresponding *k*-Hessian measure associated with the *k*-convex function −*u*.

**Proposition 10 .**

Let *u* ≥ 0 be such that −*u* is *k*-convex in *Ω*. Then, there is a constant *C* = *C*(*n*, *k*) > 0 such that if *μ* = *F*_*k*_[−*u*] then

(17) $$\begin{array}{c} CW_{2k/\left(k+1\right),k+1}^{R/8}\mu \left(\;x\;\right)\leq u\left(\;x\;\right), \end{array}$$

whenever the ball *B*_3*R*_ ⊂ *Ω*.

## 3. Proof of Theorems 1 and 2

In this section, we will give the proof of our main theorem. Firstly, we prove the following integral estimate for solutions of quasilinear equations (1), which shows that if (1) has a nontrivial *p*-superharmonic supersolution, then *ω* is absolutely continuous with respect to *σ*. The fact can be used to obtain a characterization of removable singularities for the homogeneous quasilinear equation:

(18) −Δpu=σPu,u≥0,  u  is  p-superharmonic  in  Ω,

in terms of Hausdorff measures. For more details, see Theorem  2.18 in [4] and Theorem  3.1 in [3].

**Lemma 11 .**

Let *σ* and *ω* be locally finite nonnegative measures on *Ω* and *p* > 1. There exists a constant *C* = *C*(*N*, *p*, *α*, *β*, *ℓ*) > 0 such that if *u*(*x*) is a solution to (1), then

(19) ∫BRPl,α,βudσ+ωBR≤CσBRp−1/lβ−p+1RlβN−p/lβ−p+1,

for all balls *B*_*R*_ such that *B*_2*R*_ ⊂ *Ω*.

**Proof**

Define

(20) $$\begin{array}{c} d\mu =\sigma P_{l,\alpha ,\beta }\left(\;u\;\right)+\omega . \end{array}$$

According to Proposition 6 and the definitions of *P*_*ℓ*,*α*,*β*_, we know that, for all *x* ∈ *B*_*R*_,

(21) αll!μBRRN−plβ/p−1Pl,α,βμBRxRN−p1/p−1≤CPl,α,βux.

Integrating both sides of (21) against *dσ* over *B*_*R*_, we find

(22) $$\begin{array}{c} \frac{\alpha^{l}}{l!}\sigma \left(\;B_{R}\;\right){\left[\;\frac{\mu \left(B_{R}\right)}{R^{N-p}}\;\right]}^{l\beta /\left(p-1\right)}\leq C\int_{B_{R}}P_{l,\alpha ,\beta }\left(\;u\;\right)\left(\;x\;\right)d\sigma , \end{array}$$

which combined with (20) implies that

(23) $$\begin{array}{c} \frac{\alpha^{l}}{l!}\sigma \left(\;B_{R}\;\right){\left[\;\frac{\mu \left(B_{R}\right)}{R^{N-p}}\;\right]}^{l\beta /\left(p-1\right)}\leq C\mu \left(\;B_{R}\;\right). \end{array}$$

This inequality is equivalent to

(24) $$\begin{array}{c} \mu \left(\;B_{R}\;\right)\leq C\sigma {\left(\;B_{R}\;\right)}^{\left(p-1\right)/\left(l\beta -p+1\right)}R^{l\beta \left(N-p\right)/\left(l\beta -p+1\right)}, \end{array}$$

which, together with (20), leads to (19).

**Proof of Theorem 1.**

For fixed *x*_0_ ∈ *Ω*, let *R* > 0 be such that *B*_4*R*_(*x*_0_) ⊂ *Ω*. Suppose that *u* is a positive solution of (1). In view of the lower pointwise potential estimate (14), we find that, for all *x* ∈ *B*_*R*_(*x*_0_),

(25) uxCW1,pRωx,uxCW1,pRPl,α,βudσx≥CW1,pRαll!ulβdσx=Cαll!1/p−1W1,pRulβdσx,

where *C* depends on *N*, *p*.

Restrict the integration *σ* on *B*_*R*_(*x*_0_) and let *dσ*′ = *χ*_*B*_*R*_(*x*_0_)_ *dσ*; thus, taking into account (25), we obtain

(26) ux≥Cαll!1/p−1W1,pRCW1,pRωlβdσx=C1+lβ/p−1αll!1/p−1∫0R1tN−p·∫Btx∩Brx0W1,pRωylβdσy1/p−1dtt,

in view of

(27) W1,pRωyC0N,pχBrx0y·∫rR/2ωBsx0sN−p1/p−1dss,

which combined with (26) leads to the fact that, for all *x* ∈ *B*_*R*_(*x*_0_),

(28) uxC1+lβ/p−1αll!1/p−1C0Mx0,ωlβ/p−1·W1,pRχBRx0dσx,

where *M*(*x*_0_, *ω*) is defined as

(29) $$\begin{array}{c} M\left(\;x_{0},\omega \;\right)=\overset{R/2}{\underset{r}{\int }}{\left(\;\frac{\omega \left(B_{s}\left(x_{0}\right)\right)}{s^{N-p}}\;\right)}^{1/\left(p-1\right)}\frac{ds}{s}. \end{array}$$

Thus, taking into account (26) and (28) and arguing by induction, we find

(30) ux0≥CN,p,α,β,lMx0,ωlβ/p−1nMn1x0,

where *𝔐* is a nonlinear integral operator defined by *𝔐f* = **W**_1,*p*_^*R*^(*f* ^*ℓβ*^ *dσ*′). The iterates of *𝔐* are denoted by *𝔐* ^*i*^ *f* = *𝔐*(*𝔐* ^*i*−1^ *f*). It is then easy to see from Proposition 8 that, for all *s* > 0,

(31) M1x0=W1,pRdσ′≥ϕy,Mϕsp−1y≥ϕslβ+1yslβ+1,

where *ϕ*(*y*) appears in Proposition 8. Consequently,

(32) Mn1x0≥∏j=1n−1∑i=1jlβp−1i−lβ/p−1n−j−1·ϕx0∑i=0n−1lβ/p−1i,

and this yields

(33) limsupn→∞⁡Mn1x0p−1/lβn≥CN,p,l,α,βϕx0p−1/lβ−p+1.

Here, we use the fact that

(34) $$\begin{array}{c} \overset{\infty }{\underset{i=1}{\sum }}{\left[\;\frac{p-1}{l\beta }\;\right]}^{i}=\frac{p-1}{l\beta -p+1},\;\text{if }l\beta >p-1. \end{array}$$

In the following, we will divide the proof into two cases.

*Case  1* (*u*(*x*_0_) < *∞*). In this case, (33), combined with (30), implies that

(35) $$\begin{array}{c} 1\geq C\left(\;N,p,l,\alpha ,\beta \;\right)M\left(\;x_{0},\omega \;\right){\left(\;\phi \left(\;x_{0}\;\right)\;\right)}^{\left(p-1\right)/\left(l\beta -p+1\right)}. \end{array}$$

Recalling that,

(36) ϕx0∑j=0∞cj=∑j=0∞CσBr2−jx0r2−jN−p1/p−1≥C2N,p∫0rσBtx0tN−p1/p−1dtt,

which leads to (7).

*Case  2* (*u*(*x*_0_) = *∞*). According to [12], we know that *u* < *∞* a.e. in *Ω*. Therefore, choose a sequence {*x*_*n*_}_*n*≥1_ ⊂ *B*_*R*/10_(*x*_0_), such that lim_*n*→*∞*_ *x*_*n*_ = *x*_0_ and *u*(*x*_*n*_) < *∞*. Then, (7) holds with *x*_*n*_ in place of *x*_0_, for all *n* ≥ 1. Then, (7) holds by the lower semicontinuity of Wolff potentials inequality.

The proof of inequality (8) is completely similarly and more details are omitted.

**Proof of Theorem 2.**

Let *u* be a nonnegative *p*-superharmonic of (1). Then, *u* satisfies (7), while it is well known that

(37) $$\begin{array}{c} \overset{r}{\underset{0}{\int }}{\left[\;\frac{\sigma \left(B_{t}\right)}{t^{N-p}}\;\right]}^{1/\left(p-1\right)}\frac{dt}{t}=\infty , \end{array}$$

Provided that *dσ* = |*x*|^−*r*^ *dx* with *r* > *p*, which contradicts (7).

## 4. Proof of Theorems 3 and 4

In this section, we will prove Theorem 3. It is interesting to note that, in order to prove this theorem, we should give a new iterative process.

**Proof of Theorem 3.**

This proof will be divided into two parts according to the value of *p*.

*Case  1* (1 < *p* ≤ 2). For nonnegative measurable functions *f*, define

(38) $$\begin{array}{c} N\left(\;f\;\right)\left(\;x\;\right)=W_{1,p}^{R}\left(\;f^{p-1}d\sigma^{\prime }\;\right)\left(\;x\;\right). \end{array}$$

Obviously, *𝒩* is a homogeneous superlinear operator acting on nonnegative functions. Assume that *u* is a solution of (1); then, for all *x* ∈ *B*_*R*_(*x*_0_),

(39) uxCW1,pRup−1dσ′x+CW1,pRωx=CNux+CW1,pRωx≥C2NW1,pRωx+CW1,pRωx,

where *C* depends on *N*, *p*.

Iterating (39) *n* times yields

(40) $$\begin{array}{c} u\left(\;x\;\right)\geq \overset{i=n}{\underset{i=1}{\sum }}C^{i+1}N^{i}\left(\;W_{1,p}^{R}\left[\;\omega \;\right]\;\right)\left(\;x\;\right)+CW_{1,p}^{R}\left[\;\omega \;\right]\left(\;x\;\right). \end{array}$$

Here, we use the fact that *𝒩* is a homogeneous superlinear operator and *i*th iterate of *𝒩* is defined by *𝒩* ^*i*^(*u*) = *𝒩*(*𝒩* ^*i*−1^(*u*)) for *i* > 1.

In the following, we will estimate the iterates of *𝒩*. Recall *dσ*′ = *χ*_*B*_*R*_(*x*_0_)_ *dσ*; thus,

(41) NW1,pRωx=∫0R1tN−p·∫Btx∩Brx0W1,pRωyp−1dσy1/p−1dtt,

in view of

(42) $$\begin{array}{c} W_{1,p}^{R}\left[\;\omega \;\right]\left(\;y\;\right)\geq C_{0}\left(\;N,p\;\right)M\left(\;x_{0},\omega \;\right), \end{array}$$

where *M*(*x*_0_, *ω*) is defined in (29). Consequently, for all *x* ∈ *B*_*R*_(*x*_0_),

(43) NW1,pRωx≥C0Mx0,ωW1,pRχBRx0dσx≥C0Mx0,ωϕy,

where *ϕ*(*y*) appears in Proposition 8. Obviously,

(44) $$\begin{array}{c} N^{i}\left(\;W_{1,p}^{R}\left[\;\omega \;\right]\;\right)\left(\;x\;\right)\geq {\left[\;C_{0}M\left(\;x_{0},\omega \;\right)\;\right]}^{i}\frac{\phi^{i}\left(y\right)}{i!}. \end{array}$$

Here, the following fact has been used in this inequality:

(45) $$\begin{array}{c} N\left(\;\phi^{s\left(p-1\right)}\;\right)\left(\;y\;\right)\geq \frac{\phi^{s\left(p-1\right)+1}\left(y\right)}{s\left(p-1\right)+1}. \end{array}$$

Note that *i* here is arbitrary; this fact, together with (40) and (44), leads to

(46) ux0∑i=0i=∞CC0Mx0,ωϕyii!=exp⁡CC0Mx0,ωϕy,

which, combined with (36), leads to (9) provided that *u*(*x*_0_) < *∞*. In a similar way, we can prove (9) if *u*(*x*_0_) = *∞*; more details are omitted.

*Case  2* (*p* > 2). A point worth emphasizing is that the operator *𝒩* defined by (38) does not fall within this framework since it is not a superlinear operator. Therefore, define

(47) $$\begin{array}{c} N\left(\;f\;\right)\left(\;x\;\right)={\left(\;W_{1,p}^{R}\left(\;fd\sigma^{\prime }\;\right)\;\right)}^{p-1}\left(\;x\;\right). \end{array}$$

In this case, we have

(48) uxp−1Cp−1W1,pRωp−1x≥CC0Mx0,ωp−1,uxCW1,pRup−1dσx+CW1,pRωx.

Thus, by Minkowski's inequality,

(49) uxp−1Cp−1W1,pRup−1dσp−1x+CW1,pRωp−1=Cp−1Nup−1x+CW1,pRωp−1x≥C2p−1NW1,pRωp−1x+CW1,pRωp−1,

where *C* depends on *N*, *p*. It is clear that

(50) NW1,pRωp−1x=∫0R1tN−p·∫Btx∩Brx0W1,pRωp−1dσy1/p−1dttp−1≥C0Mx0,ωW1,pRχBRx0dσp−1x≥C0Mx0,ωϕyp−1,

where *C*_0_, *M*(*x*_0_, *ω*) appears in (29). Using (49) and (50), we find

(51) uxp−1≥C2C0Mx0,ωϕyp−1+CC0Mx0,ωϕyp−1.

Therefore,

(52) $$\begin{array}{c} u{\left(\;x\;\right)}^{p-1}\geq \overset{i=\infty }{\underset{i=0}{\sum }}{\left[\;\frac{{\left[C^{2}C_{0}M\left(x_{0},\omega \right)\phi \left(y\right)\right]}^{i}}{i!}\;\right]}^{p-1}. \end{array}$$

By reverse Hölder inequality, we get

(53) ux∑i=0i=∞C2C0Mx0,ωϕyii!=exp⁡C2C0Mx0,ωϕy.

The following proof is similar to that of (46), so it is clear.

This finishes the proof of Theorem 3.

The proof of Theorem 4 is standard and will be omitted.

## 5. A Fully Nonlinear Analogue: The *k*-Hessian

We now move to *k*-Hessian operator and present fully nonlinear counterparts of the results obtained in the previous theorems. More precisely, consider fully nonlinear *k*-Hessian operator *F*_*k*_, introduced by Trudinger and Wang [15–17]:

(54) $$\begin{array}{c} F_{k}\left[\;-u\;\right]=\sigma P\left(\;u\;\right)+\omega ,\;u\geq 0,\;\;-u\text{ is }k\text{-convex in }\Omega , \end{array}$$

where *F*_*k*_[*u*] denotes the *k*-Hessian (*k* = 1,2,…, *n*),

(55) $$\begin{array}{c} F_{k}\left[\;u\;\right]=\overset{}{\underset{1\leq i_{1}<i_{2}<\cdots <i_{k}\leq n}{\sum }}\lambda_{i_{1}}\cdots \lambda_{i_{k}}, \end{array}$$

where *λ*_*i*_1__ ⋯ *λ*_*i*_*k*__ are the eigenvalues of the Hessian matrix *D* ^2^ *u*; that is, *F*_*k*_[*u*] is the sum of the *k* × *k* principal minors of *D* ^2^ *u*, which coincides with the Laplacian *F*_1_[*u*] = Δ_*p*_ *u* if *k* = 1, and the Monge-Ampère operator *F*_*n*_[*u*] = det⁡(*D* ^2^ *u*) if *k* = *n*.

The proof of the following theorems is completely analogous to that of (1). One only needs to use Propositions 9 and 10 in place of Propositions 6 and 7, respectively, and argue as in Sections 3 and 4 with **W**_2*k*/(*k*+1),*k*+1_^*R*^ in place of **W**_1,*p*_^*R*^. Therefore, the proof is omitted.

**Theorem 12 .**

Let *u*(*x*) be a solution of (54) in *Ω* with *p* > 1 and *ℓβ* > *k*. Suppose that *B*_4*R*_(*x*_0_) ⊂ *Ω*. Then, there exists a constant *M* = *M*(*N*, *p*, *α*, *β*, *ℓ*) such that

(56) ∫0rσBttN−p1/kdttk/lβ−k·∫rR/16ωBtx0tN−p1/kdtt≤M,∫0rσBttN−p1/kdttk/lβ−k·∫rR/16∫BtPl,α,βudσtN−p1/kdtt≤M,

for all 0 < *r* ≤ *R*/32.

**Theorem 13 .**

Let *u*(*x*) be a solution of (54) in *Ω* with *p* > 1 and *P*(*u*) = *σu* ^*p*−1^. Suppose that *B*_4*R*_(*x*_0_) ⊂ *Ω*. Then, there exists a constant *M* = *M*(*N*, *p*, *α*, *β*, *ℓ*) such that

(57) ∫0rσBttN−p1/kdtt∫rR/16ωBtx0tN−p1/kdtt≤M,∫0rσBttN−p1/kdtt∫rR/16∫Btup−1dσtN−p1/kdtt≤M,

for all 0 < *r* ≤ *R*/32.
